# S100A8/A9 in Myocardial Infarction: A Promising Biomarker and Therapeutic Target

**DOI:** 10.3389/fcell.2020.603902

**Published:** 2020-11-12

**Authors:** ZhuLan Cai, Qingwen Xie, Tongtong Hu, Qi Yao, Jinhua Zhao, Qingqing Wu, Qizhu Tang

**Affiliations:** ^1^Department of Cardiology, Renmin Hospital of Wuhan University, Wuhan, China; ^2^Hubei Key Laboratory of Metabolic and Chronic Diseases, Wuhan, China

**Keywords:** S100A8/A9, cardiovascular diseases, myocardial infarction, ischemia/reperfusion, biomarker, therapeutic target

## Abstract

Myocardial infarction (MI), the main cause of cardiovascular-related deaths worldwide, has long been a hot topic because of its threat to public health. S100A8/A9 has recently attracted an increasing amount of interest as a crucial alarmin that regulates the pathogenesis of cardiovascular disease after its release from myeloid cells. However, the role of S100A8/A9 in the etiology of MI is not well understood. Here, we elaborate on the critical roles and potential mechanisms of S100A8/A9 driving the pathogenesis of MI. First, cellular source of S100A8/A9 in infarcted heart is discussed. Then we highlight the effect of S100A8/A9 heterodimer in the early inflammatory period and the late reparative period of MI as well as myocardial ischemia/reperfusion (I/R) injury. Moreover, the predictive value of S100A8/A9 for the risk of recurrence of cardiovascular events is elucidated. Therefore, this review focuses on the molecular mechanisms of S100A8/A9 in MI pathogenesis to provide a promising biomarker and therapeutic target for MI.

## Introduction

Myocardial infarction (MI), a universal public health issue with high mortality and morbidity rates worldwide, seriously threatens the lives and health of people and contributes to expensive medical expenditures (Reed et al., 2017). Acute MI (AMI) is identified as myocardial necrosis that begins with acute and persistent coronary ischemia and hypoxia (Heusch and Gersh, 2017). AMI mostly occurs in the context of coronary atherosclerosis, and it manifests when the coronary atherosclerotic plaque ruptures due to changes in physiological and hemodynamic factors, such as high local wall stress and low endothelial shear stress (Ahmadi et al., 2019). Platelets and other components accumulate on the surface of the ruptured plaque, causing thrombosis. Once a developing thrombus completely or partially obstructs the coronary artery, the blood and energy supply to the heart becomes restricted. The pathogenesis of MI involves mitochondrial calcium overload, increase in hydrogen ion concentration, cellular ATP depletion, dysregulation of inflammatory cytokines, and oxidative stress (Kalogeris et al., 2016). Therefore, in the process of exploring the pathophysiological and molecular mechanism of MI, identifying new markers for early diagnosis of MI, evaluating the prognosis, and guiding new therapeutic strategies have become current research hotspots.

There is growing experimental and clinical evidence that the S100 protein family has been implicated in the occurrence and development of multiple cardiovascular diseases, and these proteins are expected to become new targets for prevention (Gonzalez et al., 2020). For example, serum S100A levels increase after acute ischemic events, are highly sensitive in myocardial injury, and may be a candidate biomarker for the diagnosis of AMI (Aydin et al., 2019). At present, a growing body of evidence has illustrated that S100A8 and its heterodimeric partner S100A9 in the S100 family are expected to not only be biomarkers to evaluate the prognosis of patients with MI (Morrow et al., 2008) but also provide new ideas for patient treatment, opening a novel field of MI research. Based on accumulating experimental evidence, S100A8/A9 also functions as an important regulator of other cardiovascular disorders, such as hypertension (Wu et al., 2014), viral myocarditis (Muller et al., 2017), autoimmune myocarditis (Otsuka et al., 2009; Muller et al., 2020), doxorubicin-induced cardiotoxicity (Pei et al., 2016), cardiac hypertrophy (Wei et al., 2015), and sepsis-associated cardiomyopathy (Boyd et al., 2008).

In the present review, we focus on relevant experimental and clinical studies concerning the roles and mechanisms by which S100A8/A9 affects the pathophysiology of MI to provide valid evidence for clinical applications in the future.

## S100A8 and S100A9 Proteins: Important Members of the S100 Protein Family

### The S100 Protein Family

The S100 proteins were named because of their ability to dissolve in 100% neutral saturated ammonium sulfate solution and belong to a unique class of non-ubiquitous small molecular weight (10–12 kDa) (Bresnick et al., 2015), Ca^2+^-modulated proteins, which were first identified in nervous system by Moore (1965). The S100 family currently has 25 known different members (including S100A1-16, S100G, S100P, and S100B), which are highly similar in sequence and structure (Gonzalez et al., 2020), and they all contain calcium-binding protein motifs (Bresnick et al., 2015). Schafer and Heizmann (1996) reported that S100 proteins consist of two distinct helix-loop-helix elongation factor (EF) hands flanked by conserved hydrophobic regions at the C- and N-terminal and connected by a central hinge region. The N-terminus usually forms a loop structure composed of 14 amino acids and has a low affinity for Ca^2+^, while the C-terminus is defined as the typical Ca^2+^-binding loop, which contains 12 amino acids and has a high affinity for Ca^2+^ (Schafer and Heizmann, 1996). S100 proteins undergo pronounced conformational changes upon binding of Ca^2+^ to the EF-hand, exposing a hydrophobic patch and facilitating discrimination of a variety of different target receptors and proteins (Donato, 2003; Santamaria-Kisiel et al., 2006).

The S100 family of proteins is encoded by their respective genes, which are highly conserved among different species (Marenholz et al., 2004). Despite the fact that S100 proteins are only expressed in vertebrates, each protein exhibits a unique pattern of tissue-specific and cell-specific expression. Furthermore, the expression of S100 proteins is strictly orchestrated for the purpose of ensuring the maintenance of immune homeostasis (Donato, 2003; Zimmer et al., 2013).

Unlike calmodulin, a kind of Ca^2+^-binding protein, which only exerts intracellular effects to regulate biological activity, some S100 proteins serve both as extracellular signaling proteins and intracellular mediators, thereby participating in multiple intracellular and/or extracellular regulatory processes in either an autocrine or paracrine manner (Donato, 2001; Donato et al., 2013). It has been reported that several extracellular S100 proteins function as damage-associated molecular patterns (DAMPs) by binding to a series of membrane receptors, such as Toll-like receptor-4 (TLR-4), receptor for advanced glycation end products (RAGE), and G-protein coupled receptors (Goyette and Geczy, 2011; Pruenster et al., 2016), participating in the control of diverse physiological functions, including proliferation, differentiation, migration/invasion, inflammation, oxidative stress, Ca^2+^ homeostasis, cell apoptosis, glycogen phosphorylation, and aggregation of macrophages (Donato et al., 2013).

### Expression and Structural Characteristics of S100A8/A9

S100A8 and S100A9 proteins are important members of the S100 family and are also named myeloid-related protein 8 (MRP8) and MRP14, respectively, due to their abundance in cells of myeloid origin (Odink et al., 1987). These proteins have unique expression profiles in different types of cells; for example, these proteins are present in some innate immune cells, including dendritic cells, neutrophils, and monocytes (Averill et al., 2011). S100A8 and S100A9 constitute approximately 40% of neutrophil cytosolic proteins and approximately 1% of monocyte cytosolic proteins (Hessian et al., 1993). However, upon activation under certain conditions, expression of these proteins is also induced in fibroblasts (Rahimi et al., 2005), mature macrophages (Ingersoll et al., 2010), vascular endothelial cells (McCormick et al., 2005; Yao and Brownlee, 2010; Landers-Ramos et al., 2017), vascular smooth muscle cells (Inaba et al., 2009), keratinocytes (Grimbaldeston et al., 2003), epithelial cells, and osteoclasts (Ehrchen et al., 2009).

The S100A8 and S100A9 genes, along with many other S100 protein genes, are located on chromosome 1q21 and form the S100 gene cluster, which is a hot spot for chromosome changes (Lesniak, 2011). It has been reported that the deletion or mutation of chromosomes in this region is closely related to the occurrence of cancer (Bresnick et al., 2015). The human S100A8 gene encodes a small molecule protein composed of 93 amino acids with a molecular mass of 10.8 kDa, while the human S100A9 gene encodes a protein consisting of 113 amino acids with a molecular mass of 13.2 kDa (Zou et al., 2013; Wang et al., 2018). S100A8 and S100A9 exist in several forms and can be homodimers, heterodimers, and tetramers (Leukert et al., 2005, 2006; Schiopu and Cotoi, 2013). Due to the poor stability of homodimers, these proteins preferentially form S100A8/A9 heterodimer complexes, which are the most dominant forms of these proteins under physiological conditions (Donato et al., 2013). Additionally, S100A9 exerts a significant effect in maintaining the stability of the S100A8 protein (Hobbs et al., 2003; Manitz et al., 2003). S100A8 and S100A9 form non-covalently linked polymers (S100A8/A9), also known as calprotectin, which have been demonstrated to possess pleotropic biological properties (Vogl et al., 2006). When Ca^2+^ is at an appropriate concentration, S100A8/A9 heterodimers polymerize to form (S100A8/A9)_2_ tetramers that are essential for biological activities (Leukert et al., 2006).

### Biological Functions of S100A8/A9

Targeted deletion of the S100A8 gene in mice is lethal (Passey et al., 1999), suggesting the significance of S100A8 in early embryonic development, while mice that are genetically deficient in S100A9 have a generally healthy phenotype but do not express S100A8 proteins (Hobbs et al., 2003; Manitz et al., 2003). Consequently, it is accepted that S100A9-knockout (KO) mice are available as functional S100A8 and S100A9 double-KO mice.

Intracellular S100A8/A9 has been considered to potentiate NADPH oxidase activity by interacting with p47phox and p67phox, two important cytosolic factors of the NADPH complex (Doussiere et al., 2001, 2002). Moreover, the formation of (S100A8/A9)_2_ tetramers in a calcium-dependent manner promotes tubulin polymerization and microtubule formation (Leukert et al., 2006). The reorganization of the microtubules, which is a prerequisite for the successful transendothelial migration of phagocytes, is regulated by S100A9 phosphorylation via the p38 mitogen-activated protein kinase (MAPK) signaling pathway (Vogl et al., 2004). Accordingly, intracellular S100A8/A9 acts a pivotal part in regulating the migration of phagocytes.

S100A8/A9 is also actively secreted or passively released from specific cells. It is specifically released by activated phagocytes in the context of inflammation during the interaction between phagocytes and activated endothelial cells (Frosch et al., 2000; Pruenster et al., 2015). The secretion of S100A8/A9 lacks signal peptides that are essential for the classic endoplasmic reticulum/Golgi pathway (Rammes et al., 1997) but seems to depend on reactive oxygen species (ROS) and K+ exchanges through ATP-sensitive K(+) channels (Tardif et al., 2015). In contrast, passive release is usually an outcome of neutrophil death (Voganatsi et al., 2001), either by necrosis or NETosis (Urban et al., 2009), which requires the production of ROS by NADPH oxidase (Fuchs et al., 2007). Innate immune cells, such as neutrophils and monocytes, express PRRs on their surface, which bind to pathogen-associated molecular patterns (PAMPs) or damage-associated molecular patterns (DAMPs) under diverse pathological conditions, inducing intracellular activation of numerous signal cascades (Newton and Dixit, 2012). Extracellular S100A8/A9 functions as a DAMP to activate RAGE (Ehlermann et al., 2006; Boyd et al., 2008) and TLR-4 (Vogl et al., 2007; Ehrchen et al., 2009), thereby acting as a potent stimulator of the innate immune response in a diversity of diseases.

Extracellular S100A8/A9 is an active mediator of various inflammatory conditions. Monocytes and macrophages treated with S100A8/A9 are able to secrete inflammatory cytokines such as tumor necrosis factor α (TNFα), and amplification of the proinflammatory response is mediated by activation of the NF-κB and p38 MAPK pathways (Sunahori et al., 2006). A genome-wide comparative bioinformatics analysis of gene expression in monocytes stimulated with S100A8 revealed overexpression of specific functional clusters related to the inflammatory response, leukocyte activation, cell migration, and the NF-κB signal transduction pathway (Fassl et al., 2015). Recombinant S100A9 induces the transcription of proinflammatory mediators in macrophages, and the response can be blocked by a RAGE antagonist or S100A9 silencing (Kawakami et al., 2020).

In addition to its proinflammatory effects, S100A8/A9 may have significant regulatory effects on inflammation as well. For example, treatment of phagocytes with S100A8/A9 for 24 h can induce a state of hyporesponsiveness in a TLR-4-dependent manner (Austermann et al., 2014). S100A8/A9 also inhibits the maturation and antigen-presenting capabilities of dendritic cells (DCs), leading to a decreased T cell response and thus avoiding excessive adaptive immune responses (Petersen et al., 2013). Surprisingly, S100A8/A9 intensifies the population and activity of myeloid-derived suppressor cells (MDSCs), a heterogeneous group of cells that are immunosuppressive during many pathological conditions (Veglia et al., 2018). S100A8/A9 can disrupt overexpansion of the neonatal-specific inflammatory monocyte population, thus protecting against death from septic shock (Heinemann et al., 2017).

## S100A8/A9 in MI

The pathological process of MI is dynamic, including two successive stages: the early inflammatory stage and later the reparatory stage. The effective improvement of cardiac performance and a good patient prognosis depend on a satisfactory balance between these two phases (Prabhu and Frangogiannis, 2016). Hence, any immunomodulatory intervention in MI has to reach the appropriate balance between successful remission of excessive inflammatory responses and reasonable preservation of reparatory regulation.

In response to ischemic injury, neutrophil recruitment immediately increased within 6 h and peaked within 24 h during a 15-day observation period (Sreejit et al., 2020). Monocytes at the infarct site represent bi-phasic participation identified by early recruitment of proinflammatory Ly6C^hi^ cells, which peak at day 3 and exert phagocytic and proteolytic functions, and late accumulation of anti-inflammatory Ly6C^lo^ monocytes, which peak at day 5 and promote angiogenesis and collagen deposition (Nahrendorf et al., 2007; Dutta and Nahrendorf, 2015; Sreejit et al., 2020). The early perspective that neutrophils and monocytes are only implicated in the pathophysiology of the inflammatory stage has been challenged by researches indicating that these cells also orchestrate the reparative phases post-MI (Hilgendorf et al., 2014; Horckmans et al., 2017). S100A8/A9 continuously works to regulate the inflammatory and reparatory stages of MI and mainly acts in the migration and differentiation of immune cells, performing an increasingly vital role in the process of MI.

### Cellular Source of S100A8/A9 in MI

Cardiac release of inflammatory cytokines and hypoxia are traits of MI and play pivotal roles in the pathogenesis of ischemic injury (Neumann et al., 1995). Enhanced secretion of S100A8/A9 in a hypoxic environment may be mediated by hypoxia-induced factor 1 (HIF-1), as HIF-1 was reported to upregulate transcriptional levels of S100A8/A9 in prostate cancer cells by directly activating the S100A8 and S100A9 promoters (Grebhardt et al., 2012). Further confirmation of the engagement of HIF-1 in the upregulation of S100A8/A9 was derived from Ahn et al. (2014), who proposed that HIF-1 upregulates S100A8 protein levels in monocytes.

Immune cells recruited from the circulation to the heart and the cardiomyocytes (CMs) themselves are important cellular constituents dedicated to the generation of cytokines in the heart during myocardial injury. Understanding the cellular origin of S100A8 and S100A9 may elucidate the importance of the mechanism underlying the pathological process of MI. Several types of cells were considered to be the sources of S100A8 and S100A9 production and release during MI, including neutrophils, macrophages, activated platelets, endothelial cells, and so on.

Actually, Du et al. (2012) did not discover any traces of S100A8 and S100A9 at any time point in neonatal Wistar rat CMs undergoing hypoxia, which is a critical feature of ischemic myocardial injury, indicating that CMs were not the origin of the elevated serum S100A8/A9 following MI. This study was not consistent with previous research showing that hypoxia treatment directly induced S100A8/A9 expression in prostate cancer cells (Grebhardt et al., 2012). Next, we examined whether the inflammatory immune cells infiltrating the heart are the sources of these proteins. Katashima et al. (2010) reported a positive correlation between serum S100A8/A9 levels and neutrophil counts that were dynamically monitored in AMI patients. The above research was further confirmed by another study describing that blood neutrophils as the only cell population independently and strongly influenced the circulating S100A8/A9 concentration in humans (Cotoi et al., 2014). Subsequently, Katashima et al. (2010) used double immunostaining to evaluate the infarcted heart in patients with AMI and demonstrated that S100A8/A9 complex mainly colocalized with neutrophils in infarcted myocardium during the early acute phase and highly colocalized with macrophages during the subacute phase, which was consistent with the findings of Rammes et al. (1997) and Boussac and Garin (2000). Another study also revealed that the synthesis and expression of S100A8/A9 were observably elevated in macrophages isolated from human bone marrow (BM) following hypoxia-ischemia in a time-dependent manner (Shi and Yi, 2018). More recently, Sreejit et al. (2020) applied cell sorting and flow cytometry to enrich CD45^+^ leukocytes from the infarcted heart in mice induced by permanent ligation of the left anterior descending (LAD) artery and found that neutrophils were the predominant source of S100A8/A9. This discrepancy may be explained by inconsistent time points of detection. Additionally, previous studies have shown that blood constituents such as platelets from patients with acute ST-segment-elevation MI (STEMI) could be reservoirs of S100A8 and S100A9 (Healy et al., 2006; Morrow et al., 2008). However, the abundance of S100A8/A9 proteins in platelets was almost negligible in comparison with that in neutrophils after MI (Sreejit et al., 2020).

Taken together, these findings indicate that neutrophils and macrophages infiltrating the infarcted myocardium contribute significantly as the main sources of the increased S100A8/A9 concentration in cardiac tissue following AMI.

### Short-Term S100A9 Blockade Improves Cardiac Function

**FIGURE 1:**
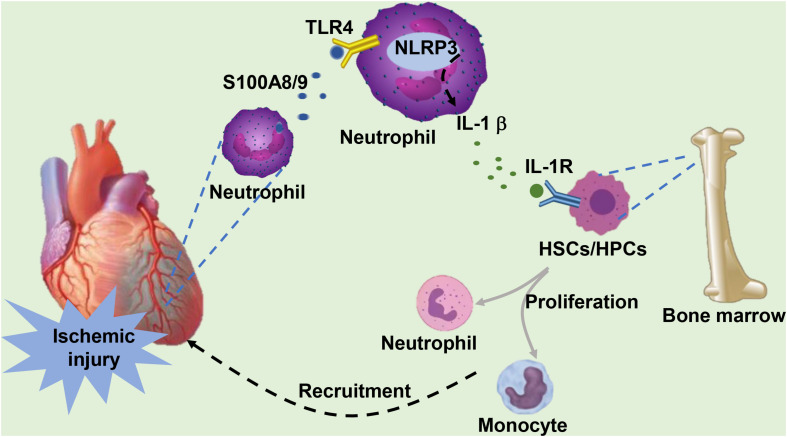
Under ischemic injury, neutrophils infiltrating the heart secrete S100A8/A9. S100A8/A9 binds to TLR-4 on the surface of cardiac or circulating neutrophils, subsequently activating the formation of downstream NLRP3 and stimulating the release of IL-1β. IL-1 binds to IL-1R on the surface of hematopoietic stem cells (HSCs) and hematopoietic progenitor cells (HPCs) in the bone marrow, which stimulates the proliferation and differentiation of HSCs into neutrophils and monocytes. These cells are eventually recruited to the infarcted heart in response to ischemic injury.

S100A8/A9 provides an important first signal for inducing the hematopoietic response in the BM by directly interacting with RAGE on common myeloid progenitor cells in diabetes (Nagareddy et al., 2013) or through TLR-4-mediated mechanisms in obesity (Nagareddy et al., 2014). In response to MI, neutrophils abundantly expressing the specific alarmin S100A8/A9 rapidly traffic to the ischemic myocardium. Neutrophil-derived S100A8/A9 subsequently interacts with TLR-4 on cardiac/circulating neutrophils, priming the nucleotide-binding oligomerization domain-like receptor protein 3 (NLRP3) inflammasome in neutrophils and facilitating the secretion of interleukin (IL) 1 (IL-1β) in murine models of MI (Sreejit et al., 2020). The released IL-1 binds to IL-1 receptor type 1 (IL-1R1) on hematopoietic stem cells (HSCs) and hematopoietic progenitor cells (HPCs) in the BM, which in turn stimulates the differentiation of these cells into granulocytes in a cell-intrinsic fashion (Sager et al., 2015). The recognition that S100A8/A9 is an upstream event in MI-induced granulopoiesis was further supported by a mouse model with targeted interference of S100A9, in which genetic deletion of S100A9 or pharmacological blockade of S100A8/A9 with ABR-215757 notably constrained granulopoiesis with decreased numbers of neutrophils and monocytes in the circulation and heart and a reduced number of granulocyte precursors in the BM, as well as apparent downregulation of NLRP3 and IL-1 β transcripts in cardiac neutrophils (Sreejit et al., 2020). In response to ischemic injury, neutrophils facilitate the proliferation and differentiation of BM-derived cells by secreting IL-1β, which is mediated by S100A8/9, and medullary granulopoiesis is activated to satisfy the excessive recruitment and demand for neutrophils in the infarcted heart (Figure 1).

S100A8/A9 is a potent stimulator of neutrophils and enhances neutrophil chemotaxis and migration (Ryckman et al., 2003; Vogl et al., 2004). Intravenous injection of S100A8/A9 stimulates massive mobilization of neutrophils from the BM into the circulation and directs their migration to the inflammatory site (Vandal et al., 2003). S100A8/A9-mediated enhanced infiltration of immune cells at the infarct area may be attributed to the effect of S100A8/A9 in regulating adhesion by upregulating the expression of the leukocyte β_*2*_ integrin Mac-1 (CD11b/CD18, αMβ2) (Bouma et al., 2004; Scott et al., 2020). The binding of S100A8/A9 to TLR-4 brings about MyD88-induced small GTPase Rap1 activation and subsequently leads to β2 integrin upregulation (Pruenster et al., 2015). Mac-1 is the most important β_*2*_ integrin on the surface of neutrophils and mediates the recruitment of neutrophils across vascular endothelial cells to the inflammation site through interacting with intercellular cell adhesion molecule-1 (ICAM-1). Thus, a positive feedback loop is formed between the generation of S100A8/A9 and the infiltration of inflammatory cells in the ischemic myocardium.

Blockade S100A9 for short-term during the first 3 days post-MI with the specific blocker ABR-238901, which inhibits the binding between S100A9 and its receptors (TLR-4 and RAGE) (Bjork et al., 2009), decreases neutrophils and macrophages infiltrating in the ischemic myocardium (Marinkovic et al., 2019). Elevated serum neutrophils and monocytes have been associated with left ventricular dysfunction, which is an independent and strong predictor of adverse prognoses in MI patients (Barron et al., 2001; Maekawa et al., 2002). Short-term blockade of S100A9 improves cardiac performance in mice with MI, as shown by a trend toward increased left ventricular ejection fraction and cardiac output, a reduced percentage of apoptotic cells and a diminished infarction scar size (Marinkovic et al., 2019). These data were further verified by work from Sreejit et al. (2020), who described the significant improvement in cardiac performance in mice transplanted with BM from S100A9−/− mice. The improvement in cardiac function and mitigation of the local and systemic inflammatory response might be ascribed to S100A9 blockade-mediated suppression of inflammatory immune cell accumulation. Additionally, the binding of S100A8/A9 to TLR-4 leads to translocation of NF-κB into the nucleus via TIR domain-containing adaptor inducing interferon-β (TRIF)-dependent pathway or myeloid differentiation factor 88 (MyD88)-dependent pathway and consequently results in upregulating production of pro-inflammatory cytokines (Pruenster et al., 2016), which further amplifies the inflammatory response. On the other hand, cluster of differentiation 68 (CD68) expressed on macrophages probably serves as a receptor-like protein for extracellular S100 proteins. S100A8 and S100A9 proteins bind to CD68 with sugar chains and form CD68-S100A8 and CD68-S100A9 complexes with the molecule, whereby affecting immune functions of macrophages via an autocrine signal transduction pathway and consequently upregulating the expression of proinflammatory cytokines, especially IL-6 and TNF-a (Okada et al., 2016). Short-term S100A9 blockade decreases cytokine expression in inflammatory macrophages and increases the expression of signature genes associated with a reparatory macrophage phenotype, providing a reparatory microenvironment in the ischemic myocardium (Marinkovic et al., 2019). S100A8/9 plays a detrimental role in the early stage of MI, and therapeutic strategies to block S100A8/A9 will benefit patients with MI.

### Extended S100A9 Blockade Promote Adverse Cardiac Remodeling in MI

In striking contrast to the benefits of short-term treatment for 3 days, extended S100A9 blockade for 21 days results in the acceleration of left ventricular remodeling and progressive deterioration of cardiac function (Marinkovic et al., 2020). These contradictory findings might be explained by the different functions of S100A9 in different pathological processes of the same disease.

**FIGURE 2:**
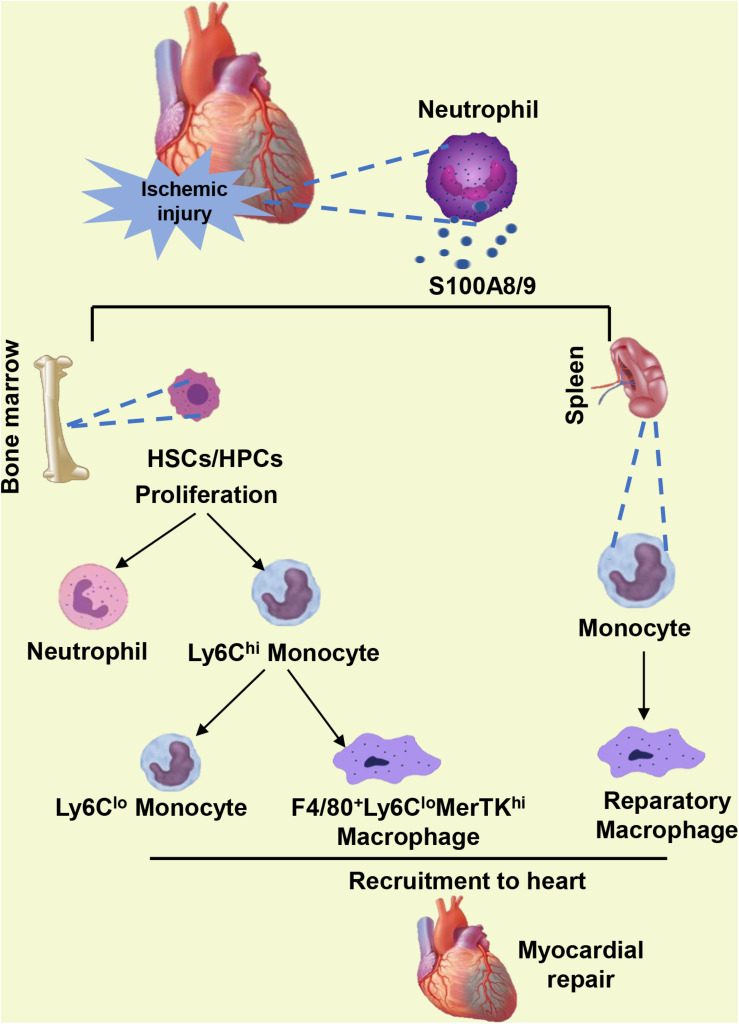
When ischemic injury occurs, neutrophils infiltrating the heart secrete S100A8/A9. S100A8/A9 facilitates the proliferation of HSCs and HPCs in the bone marrow and the differentiation of inflammatory Ly6C^hi^ monocytes into reparatory Ly6C^lo^ monocytes or reparatory F4/80^+^Ly6C^lo^MerTK^hi^ macrophages. Furthermore, S100A8/A9 in the reparatory phase also promotes the mobilization of monocytes from the splenic reservoir to the ischemic myocardium and boosts their transformation into reparatory macrophages.

The HSC subset expressing the MCP-1 receptor CCR2 has been identified to be one of the most upstream contributors to ischemic injury and has an important effect on myocardial healing (Dutta et al., 2015). However, sustained S100A9 blockade inhibits the proliferation of HSCs and HPCs in the BM, blunts the transition from CCR2^–^ to CCR2^+^ HSCs, and hampers phagocyte egress from the BM (Marinkovic et al., 2019, 2020). Post-MI, the differentiation of inflammatory Ly6C^hi^ monocytes into reparatory Ly6C^lo^ monocytes or reparatory F4/80^+^Ly6C^lo^ macrophages is mediated by the transcription factor NR4A1 (Nur77) (Hanna et al., 2011; Hilgendorf et al., 2014; Terry and Miller, 2014). The reparatory F4/80^+^Ly6C^lo^MerTK^hi^ macrophages present in the myocardium during the reparatory phase after ischemic injury clear the accumulated apoptotic CMs via efferocytosis (Wan et al., 2013). Pharmacological blockade of S100A9 in the long term leads to reduced number of reparatory macrophage populations in the ischemic heart (Marinkovic et al., 2020). The decrease in reparatory macrophages in the post-ischemic myocardium and the impairment of phenotype transition from Ly6C^hi^ to Ly6C^lo^ macrophages may be explained by S100A9 blockade-mediated Nur77 inhibition in mice, as S100A9 upregulates the expression and activity of Nur77 (Marinkovic et al., 2020). Furthermore, the continuous blockade of S100A9 into the reparatory phase also inhibits the mobilization of monocytes from the splenic reservoir to the ischemic myocardium and hinders their transformation into reparatory macrophages (Marinkovic et al., 2020) (Figure 2). The role of S100A9 in the repair period of MI was further confirmed by another experiment in which a reduction in infiltrating monocytes and macrophages in the myocardium with an approximately 50% decrease in the number of reparatory Ly6C^lo^MerTK^hi^ macrophages was detected in S100A9 gene-deficient mice (Marinkovic et al., 2020). Additionally, S100A8/S100A9 complex binds to cluster of differentiation 69 (CD69) in monocyte and forms CD69-S100A8/S100A9 association which positively regulates Treg-cell differentiation through the inhibition of signal transducer and activator of transcription 3 (STAT3) signaling by upregulation of suppressors of cytokine signaling 3 (SOCS3) expression (Lin et al., 2015). This function of S100A8/S100A9 complex likely causes immunosuppression of many immune cells and avoids excessive immune response. However, excessive blockade of S100A8/A9 probably abolish this beneficial effect during the reparatory phase of MI.

Long-term S100A9 blockade with ABR-238901 in mice with myocardial ischemia results in progressive left ventricular remodeling and gradual deterioration of cardiac function, indicating additional cardioprotective effects of S100A8/A9 by polarizing macrophages toward a reparative phenotype during the recovery phase post-MI. These data support customized therapeutic strategies for short-term anti-S100A9 blockade during the early inflammatory phase post-MI, while long-term blockade affects the recovery of cardiac function during the reparatory phase; therefore, it is necessary to determine an appropriate therapeutic window.

### S100A8/A9 Aggravates Ischemia/Reperfusion (I/R) Injury

Currently, the reperfusion strategy is widely accepted as an effective and standard therapy for STEMI (Vogel et al., 2019). Reperfusion can paradoxically protect the ischemic heart from developing myocardial necrosis, but it also initiates a series of cascade reactions that can exacerbate and prolong post-ischemic injury (Hausenloy and Yellon, 2016). The inflammatory response (Marinkovic et al., 2019), oxidative stress (Ferrari et al., 2004), and mitochondrial dysfunction (Walters et al., 2012) have long been regarded as critical pathophysiological phenomena contributing to cardiac structural and functional alterations after ischemia/reperfusion (I/R) injury.

A seminal paper by Li et al. (2019) identified that S100A8/A9 expression was sustained in the heart in mice after I/R throughout the early stage of I/R, peaking at 6 h after reperfusion and returning to baseline at day 7, as determined by time-series transcriptomics analysis. To fully understand the mechanism of S100A8/A9 in I/R, it is also important to clarify the cellular sources of S100A8/A9 during reperfusion injury. Li et al. (2019) found that CXCR2^+^ neutrophils were the major sources of S100A8/A9 secretion, as the dynamic changes in CXCR2^+^ neutrophil infiltration of the heart during I/R were consistent with the pattern of S100A8/A9 expression, which were further confirmed by the fact that cardiac S100A8/A9 expression was substantially reduced in CXCR2-KO mice. Chemokine (C-X-C motif) ligand 1 (CXCL1), a specific chemokine for CXCR2, is responsible for the recruitment of neutrophils expressing the chemokine receptor CXCR2 into the inflammatory microenvironment (Drummond et al., 2019).

Loss-of function and gain-of-function studies further demonstrated the significant function of S100A8/A9 in cardiac I/R. Ubiquitous S100A9 KO mice exhibited a simultaneous deletion of S100A8 and S100A9 (Cheng et al., 2008), probably owing to increased degradation of S100A8 protein in the loss of its dimerization partner S100A9, as S100A8 protein stability is highly dependent on the presence of S100A9 (Hobbs et al., 2003; Manitz et al., 2003). More recently, a study demonstrated that infarct size was significantly decreased, cardiac contractile function was improved, CM death was markedly reduced, and myocardial fibrosis was lessened in mice with global S100A9 deficiency following I/R (Li et al., 2019), which suggests a detrimental role of S100A9. Furthermore, a significantly reduced infarct region, increased cardiac function, and decreased myocardial fibrosis were observed post-I/R in mice treated with a S100A9 neutralizing antibody (nAb) (Li et al., 2019). Strong proof for the unfavorable effect of S100A9 in I/R injury stems from Marinkovic et al. (2019), who observed an efficient improvement in cardiac function in mice with short-term S100A9 blockade after I/R.

In contrast, transgenic mice with BM-specific overexpression of S100A9 exhibited an increase in infarct size, exacerbation of cardiac contractile function, enrichment in CM death and amplification of cardiac fibrosis (Li et al., 2019). Additional intraperitoneal treatment of mice with I/R injury with human recombinant S100A8/A9 proteins (rS100A8/A9) resulted in observably raising influx of inflammatory immune cells into the myocardial ischemic zone and extensive interstitial collagen deposition, which further promoted maladaptive myocardial remodeling (Volz et al., 2012).

**FIGURE 3:**
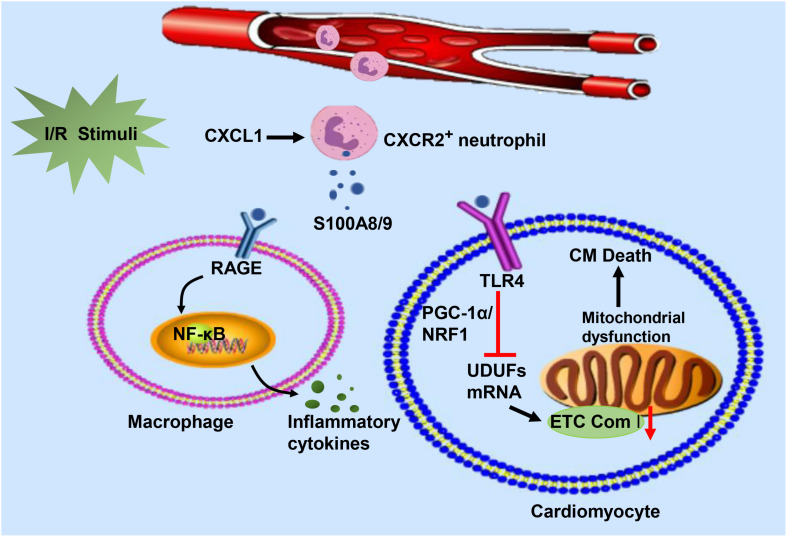
In response to I/R injury, neutrophils migrate from the circulation to the injured heart in response to CXCL and release S100A8/A9. The heterodimeric complex binds to RAGE on the surface of immune cells and stimulates immune cells to secrete inflammatory cytokines by activating the NF-κB signaling pathway. On the other hand, S100A8/A9 interacts with TLR-4 to inhibit NDUFs gene expression by suppressing the PGC-1/NRF1 signaling pathway, which decreases cardiac mitochondria electron transport chain (ETC) complex I activity. Mitochondrial dysfunction eventually leads to the death of cardiomyocytes.

It is accepted that the S100A8/A9 heterodimeric complex, a well-known extracellular ligand, interacts with TLR-4 or RAGE, inducing diverse intracellular signaling cascades (Pruenster et al., 2016). The binding of S100A8/A9 and TLR-4 activates the Erk signaling pathway and subsequently inhibits the PGC-1α/NRF1 axis, which in turn downregulates the gene transcription of mitochondrial electron transport chain (ETC) complex I subunits (NDUFs) (Li et al., 2019). Mitochondrial dysfunction leads to impaired ATP biosynthesis disorders. Insufficient ATP synthesis is particularly detrimental to the heart because it requires enough energy to maintain normal activity. Moreover, a significant upregulation of RAGE results in sustained NF-κB activation (Andrassy et al., 2006), triggering a continuous inflammatory response and generating key proinflammatory mediators. S100A8/A9 might stimulate this positive feedback loop by engaging with RAGE (Kawakami et al., 2020), driving adverse cardiac remodeling post I/R. Upon ligation with its various receptors, S100A8/A9 can regulate CM death and cytokine release from innate immune cells in response to I/R injury (Figure 3).

## Clinical Research on S100A8/A9 in MI

There are increasing experimental studies and clinical evidence indicating that S100A8/A9 may favor the development of atherosclerosis (McCormick et al., 2005; Miyamoto et al., 2008). Clinical study in acute STEMI patients has confirmed that platelet and S100A8/A9 heterodimer were colocalized in coronary artery thrombus. It was found that S100A9 binding to the scavenger receptor CD36 induces platelet activation via a signal cascade of VAV and JNK phosphorylation, thereby accelerating thrombosis (Wang et al., 2014). More interestingly, S100A8 and S100A9 protein and mRNA expression were also measured in macrophages and foam cells of atherosclerotic plaques from human carotid and aortic specimens (McCormick et al., 2005). In addition, the level of S100A9 in plaques was positively correlated with IL-6, IL-8, matrix metalloproteinase (MMP)-8, and MMP-9 levels and negatively correlated with MMP-2 concentration (Ionita et al., 2009). These factors facilitate extracellular matrix degradation; therefore, atherosclerotic plaques are prone to rupture. Hence, S100A8/A9 is closely related to atherosclerosis, which is the underlying cause and pathological foundation of MI.

Healy et al. (2006) used transcriptional profiling analysis to identify S100A8/A9 as a novel predictor of MI by examining platelet mRNA transcripts and showed that S100A9 mRNA levels in patients with STEMI were strikingly elevated compared to those of patients with stable coronary artery disease. Published data from Shi and Yi (2018) also reported that serum S100A8/A9 levels were increased in AMI patients and were more pronounced in patients with cardiac rupture. Further, Katashima et al. reported on the dynamic monitoring of circulating S100A8/A9 levels in patients with AMI and unstable angina pectoris (UAP) in the acute phase. Compared with those of UAP patients, the early circulating S100A8/A9 levels of AMI patients were lower. At 3–5 days, the circulating S100A8/A9 levels of patients with AMI were significantly higher than those of patients with UAP. The circulating S100A8/A9 levels reached a peak on days 3–5 after ischemic injury and continued to increase for several weeks after the event (Katashima et al., 2010). Additionally, a clinical study showed that compared with patients with stable angina pectoris or individuals with normal coronary artery morphology assessed by coronary angiography, circulating S100A8/A9 levels were highly elevated in patients with ACS. The occurrence of elevated plasma S100A8/A9 before classic myocardial damage markers such as myoglobin and troponin makes it a promising candidate for the diagnosis and prediction of ACS (Altwegg et al., 2007). However, a single center prospective cohort study indicated that S100A8/A9 was a poor diagnostic marker with a sensitivity of 28% for MI in patients with non-traumatic chest pain and did not offer supernumerary diagnostic value comparable to that of cardiac troponin (Vora et al., 2012).

S100A8/A9 is also valuable for predicting the risk of recurrence of cardiovascular events in patients with acute ischemia. A nested case-control study of PROVE IT-TIMI intensive lipid-lowering therapy for ACS was performed to examine follow-up of patients after 30 days of acute cardiovascular events and found that S100A8/A9 was increased in patients with end point events (MI or cardiovascular death). Patients with S100A8/A9 values in the highest quartile had 2.0-fold increased odds of developing a recurrent cardiovascular event compared with those in the lowest quartile after adjusting for high-sensitivity C-reactive protein, hypertension, diabetes, previous cardiovascular disease, and heart failure (Morrow et al., 2008). The association between plasma S100A8/A9 levels and left ventricular dysfunction was studied in a cohort of 524 ACS patients. The patients with high plasma S100A8/A9 levels within 24 h of an acute coronary event had higher risk of hospitalization for a main diagnosis of heart failure and reduced left ventricular ejection fractions at 1 year after the MI (Marinkovic et al., 2019). Recently, a study enrolled 210 STEMI patients who received percutaneous coronary intervention (PCI) within 24 h. Patients with elevated serum S100A8/A9 levels 1 day post-PCI were more likely to undergo major adverse cardiovascular events (MACEs), including cardiogenic shock, HF/rehospitalization, and cardiovascular mortality (Li et al., 2019). Furthermore, S100A8/A9 can also predict the risk of future cardiovascular events in healthy individuals. A study with a median follow-up of 16.2 years has shown that circulating S100A8/A9 levels could effectively predict the future risk of the events of coronary artery disease and cardiovascular death in a healthy female population. The circulating S100A8/A9 concentration was positively correlated with traditional cardiovascular risk factors, including glycosylated hemoglobin A1c, low-density lipoprotein, body mass index, and smoking (Cotoi et al., 2014).

Accordingly, this clinical evidence supports that S100A8/A9 is a feasible biomarker to distinguish patients with ACS from those with stable angina and that elevated circulating S100A8/A9 is clinically relevant to long-term adverse prognosis (Table 1).

**TABLE 1:** Clinic research on S100A8/A9 in MI.

Authors	Clinic studies	References
Healy et al.	S100A9 mRNA levels in patients with STEMI were elevated.	Healy et al., 2006
Shi et al.	Serum S100A8/A9 levels were increased in AMI patients and were more pronounced in patients with cardiac rupture.	Shi and Yi, 2018
Katashima et al.	The circulating S100A8/A9 levels reached a peak on days 3–5 after ischemic injury and continued to increase for several weeks after the event.	Katashima et al., 2010
Altwegg et al.	Circulating S100A8/A9 levels were elevated in patients with AMI and its occurrence before classic myocardial damage markers.	Altwegg et al., 2007
Vora et al.	A single center prospective cohort study indicated that S100A8/A9 was a poor diagnostic marker with a sensitivity of 28% for MI in patients with non-traumatic chest pain.	Vora et al., 2012
Morrow et al.	S100A8/A9 was increased in patients with end point events (MI or cardiovascular death) and S100A8/A9 was associated with a high risk of recurrence of cardiovascular events.	Morrow et al., 2008
Marinkovic et al.	The patients with high plasma S100A8/A9 levels within 24 h of an acute coronary event had higher risk of hospitalization for a main diagnosis of heart failure and reduced left ventricular ejection fractions.	Marinkovic et al., 2019
Li et al.	Patients with elevated serum S100A8/A9 levels 1 day post-PCI were more likely to undergo major adverse cardiovascular events (MACEs).	Li et al., 2019
Cotoi et al.	The circulating S100A8/A9 concentration was positively correlated with traditional cardiovascular risk factors and circulating S100A8/A9 levels could effectively predict the future risk of the events of coronary artery disease and cardiovascular death.	Cotoi et al., 2014

## Conclusion

Increased circulating S100A8/A9 levels are apparently associated with MI. However, the specific molecular mechanisms and pathways of S100A8/A9 underlying this acute event are now being elucidated. Given S100A8 KO mice are lethal and S100A9 KO mice also lack S100A8 proteins, it remains a challenge to distinguish between the specific role of S100A9 and S100A8 proteins in MI. Currently, multiple available possibilities to neutralize S100A8/A9 have been indicated in various disease models (Table 2). These studies allow us to set further objective to explore the applicability of antibodies or compounds that block the binding of S100A8/A9 to CD molecules. In addition, therapeutic strategies for short-term anti-S100A9 blockade during the early inflammatory phase post-MI can effectively mitigated post-ischemic myocardial damage, while long-term blockade may induce undesired side effects and offset the favorable consequences of short-term treatment. Therefore, how to determine an appropriate therapeutic window to achieve optimal effects of S100A9 blockade will be a hot topic with research prospects in the future.

**TABLE 2:** Currently available possibilities to neutralize S100A8/A9.

Strategy	Biological action	References
ABR-238901	Block the binding of S100A8/A9 to TLR4 and RAGE	Marinkovic et al., 2019, 2020
Tasquinimod (ABR-215050)	Block the binding of S100A8/A9 to TLR4 and RAGE	Kallberg et al., 2012; Raymond et al., 2014
Paquinimod (ABR-215757)	Block the binding of S100A8/A9 to TLR4 and RAGE	Wache et al., 2015; Kraakman et al., 2017
43/8 Antibody	Block the binding of S100A8/A9 to TLR4 and RAGE	Bjork et al., 2009
Anti-S100A9 IgG antibody	Neutralizes extracellular S100A9	Foronjy et al., 2016
S100A9 small interfering RNA	Silence S100A9 expression	New et al., 2013
Genetic ablation of S100A9	Knockout S100A8 and S100A9	Schelbergen et al., 2016; Vogl et al., 2018; Li et al., 2019
Narciclasine	Reduced the plasma levels of S100A8/A9	Kingsley et al., 2020

## Author Contributions

ZC and QT took charge of the design of the work. QX and TH were responsible for the collection of data. QY and JZ analyzed the data. ZC drafted the manuscript. ZC and QW reviewed and revised the manuscript. All authors contributed to the article and approved the submitted version.

## Conflict of Interest

The authors declare that the research was conducted in the absence of any commercial or financial relationships that could be construed as a potential conflict of interest.

## References

[B1] Ahmadi A. Argulian E. Leipsic J. Newby D. E. Narula J. (2019). From subclinical atherosclerosis to plaque progression and acute coronary events: JACC state-of-the-art review. *J. Am. Coll. Cardiol.* 74 1608–1617. 10.1016/j.jacc.2019.08.012 31537271

[B2] Ahn G. O. Seita J. Hong B. J. Kim Y. E. Bok S. Lee C. J. (2014). Transcriptional activation of hypoxia-inducible factor-1 (HIF-1) in myeloid cells promotes angiogenesis through VEGF and S100A8. *Proc. Natl. Acad. Sci. U.S.A.* 111 2698–2703. 10.1073/pnas.1320243111 24497508 PMC3932909

[B3] Altwegg L. A. Neidhart M. Hersberger M. Muller S. Eberli F. R. Corti R. (2007). Myeloid-related protein 8/14 complex is released by monocytes and granulocytes at the site of coronary occlusion: a novel, early, and sensitive marker of acute coronary syndromes. *Eur. Heart J.* 28 941–948. 10.1093/eurheartj/ehm078 17387139

[B4] Andrassy M. Igwe J. Autschbach F. Volz C. Remppis A. Neurath M. F. (2006). Posttranslationally modified proteins as mediators of sustained intestinal inflammation. *Am. J. Pathol.* 169 1223–1237. 10.2353/ajpath.2006.050713 17003481 PMC1780182

[B5] Austermann J. Friesenhagen J. Fassl S. K. Petersen B. Ortkras T. Burgmann J. (2014). Alarmins MRP8 and MRP14 induce stress tolerance in phagocytes under sterile inflammatory conditions. *Cell Rep.* 9 2112–2123. 10.1016/j.celrep.2014.11.020 25497086

[B6] Averill M. M. Barnhart S. Becker L. Li X. Heinecke J. W. Leboeuf R. C. (2011). S100A9 differentially modifies phenotypic states of neutrophils, macrophages, and dendritic cells: implications for atherosclerosis and adipose tissue inflammation. *Circulation* 123 1216–1226. 10.1161/circulationaha.110.985523 21382888 PMC3072335

[B7] Aydin S. Ugur K. Aydin S. Sahin I. Yardim M. (2019). Biomarkers in acute myocardial infarction: current perspectives. *Vasc. Health Risk Manag.* 15 1–10. 10.2147/vhrm.s166157 30697054 PMC6340361

[B8] Barron H. V. Harr S. D. Radford M. J. Wang Y. Krumholz H. M. (2001). The association between white blood cell count and acute myocardial infarction mortality in patients > or = 65 years of age: findings from the cooperative cardiovascular project. *J. Am. Coll. Cardiol.* 38 1654–1661. 10.1016/s0735-1097(01)01613-8 11704377

[B9] Bjork P. Bjork A. Vogl T. Stenstrom M. Liberg D. Olsson A. (2009). Identification of human S100A9 as a novel target for treatment of autoimmune disease via binding to quinoline-3-carboxamides. *PLoS Biol* 7:e97. 10.1371/journal.pbio.1000097 19402754 PMC2671563

[B10] Bouma G. Lam-Tse W. K. Wierenga-Wolf A. F. Drexhage H. A. Versnel M. A. (2004). Increased serum levels of MRP-8/14 in type 1 diabetes induce an increased expression of CD11b and an enhanced adhesion of circulating monocytes to fibronectin. *Diabetes* 53 1979–1986. 10.2337/diabetes.53.8.1979 15277376

[B11] Boussac M. Garin J. (2000). Calcium-dependent secretion in human neutrophils: a proteomic approach. *Electrophoresis* 21 665–672. 10.1002/(sici)1522-2683(20000201)21:3<665::aid-elps665>3.0.co;2-u 10726775

[B12] Boyd J. H. Kan B. Roberts H. Wang Y. Walley K. R. (2008). S100A8 and S100A9 mediate endotoxin-induced cardiomyocyte dysfunction via the receptor for advanced glycation end products. *Circ. Res.* 102 1239–1246. 10.1161/circresaha.107.167544 18403730

[B13] Bresnick A. R. Weber D. J. Zimmer D. B. (2015). S100 proteins in cancer. *Nat. Rev. Cancer* 15 96–109. 10.1038/nrc3893 25614008 PMC4369764

[B14] Cheng P. Corzo C. A. Luetteke N. Yu B. Nagaraj S. Bui M. M. (2008). Inhibition of dendritic cell differentiation and accumulation of myeloid-derived suppressor cells in cancer is regulated by S100A9 protein. *J. Exp. Med.* 205 2235–2249. 10.1084/jem.20080132 18809714 PMC2556797

[B15] Cotoi O. S. Duner P. Ko N. Hedblad B. Nilsson J. Bjorkbacka H. (2014). Plasma S100A8/A9 correlates with blood neutrophil counts, traditional risk factors, and cardiovascular disease in middle-aged healthy individuals. *Arterioscler. Thromb. Vasc. Biol.* 34 202–210. 10.1161/atvbaha.113.302432 24202303

[B16] Donato R. (2001). S100: a multigenic family of calcium-modulated proteins of the EF-hand type with intracellular and extracellular functional roles. *Int. J. Biochem. Cell Biol.* 33 637–668. 10.1016/s1357-2725(01)00046-2 11390274

[B17] Donato R. (2003). Intracellular and extracellular roles of S100 proteins. *Microsc. Res. Tech.* 60 540–551. 10.1002/jemt.10296 12645002

[B18] Donato R. Cannon B. R. Sorci G. Riuzzi F. Hsu K. Weber D. J. (2013). Functions of S100 proteins. *Curr. Mol. Med.* 13 24–57. 22834835 PMC3707951

[B19] Doussiere J. Bouzidi F. Vignais P. V. (2001). A phenylarsine oxide-binding protein of neutrophil cytosol, which belongs to the S100 family, potentiates NADPH oxidase activation. *Biochem. Biophys. Res. Commun.* 285 1317–1320. 10.1006/bbrc.2001.5324 11478801

[B20] Doussiere J. Bouzidi F. Vignais P. V. (2002). The S100A8/A9 protein as a partner for the cytosolic factors of NADPH oxidase activation in neutrophils. *Eur. J. Biochem.* 269 3246–3255. 10.1046/j.1432-1033.2002.03002.x 12084065

[B21] Drummond R. A. Swamydas M. Oikonomou V. Zhai B. Dambuza I. M. Schaefer B. C. (2019). CARD9(+) microglia promote antifungal immunity via IL-1beta- and CXCL1-mediated neutrophil recruitment. *Nat. Immunol.* 20 559–570. 10.1038/s41590-019-0377-2 30996332 PMC6494474

[B22] Du C. Q. Yang L. Han J. Yang J. Yao X. Y. Hu X. S. (2012). The elevated serum S100A8/A9 during acute myocardial infarction is not of cardiac myocyte origin. *Inflammation* 35 787–796. 10.1007/s10753-011-9375-8 21887593

[B23] Dutta P. Nahrendorf M. (2015). Monocytes in myocardial infarction. *Arterioscler. Thromb. Vasc. Biol.* 35 1066–1070. 10.1161/atvbaha.114.304652 25792449 PMC4409536

[B24] Dutta P. Sager H. B. Stengel K. R. Naxerova K. Courties G. Saez B. (2015). Myocardial Infarction Activates CCR2(+) hematopoietic stem and progenitor cells. *Cell Stem Cell* 16 477–487. 25957903 10.1016/j.stem.2015.04.008 PMC4426344

[B25] Ehlermann P. Eggers K. Bierhaus A. Most P. Weichenhan D. Greten J. (2006). Increased proinflammatory endothelial response to S100A8/A9 after preactivation through advanced glycation end products. *Cardiovasc. Diabetol.* 5:6. 10.1186/1475-2840-5-6 PMC1475836 16573830

[B26] Ehrchen J. M. Sunderkotter C. Foell D. Vogl T. Roth J. (2009). The endogenous Toll-like receptor 4 agonist S100A8/S100A9 (calprotectin) as innate amplifier of infection, autoimmunity, and cancer. *J. Leukoc. Biol.* 86 557–566. 10.1189/jlb.1008647 19451397

[B27] Fassl S. K. Austermann J. Papantonopoulou O. Riemenschneider M. Xue J. Bertheloot D. (2015). Transcriptome assessment reveals a dominant role for TLR4 in the activation of human monocytes by the alarmin MRP8. *J. Immunol.* 194 575–583. 10.4049/jimmunol.1401085 25505274

[B28] Ferrari R. Guardigli G. Mele D. Percoco G. F. Ceconi C. Curello S. (2004). Oxidative stress during myocardial ischaemia and heart failure. *Curr. Pharm. Des.* 10 1699–1711. 10.2174/1381612043384718 15134567

[B29] Foronjy R. F. Ochieng P. O. Salathe M. A. Dabo A. J. Eden E. Baumlin N. (2016). Protein tyrosine phosphatase 1B negatively regulates S100A9-mediated lung damage during respiratory syncytial virus exacerbations. *Mucosal Immunol.* 9 1317–1329. 10.1038/mi.2015.138 26813343 PMC4963308

[B30] Frosch M. Strey A. Vogl T. Wulffraat N. M. Kuis W. Sunderkotter C. (2000). Myeloid-related proteins 8 and 14 are specifically secreted during interaction of phagocytes and activated endothelium and are useful markers for monitoring disease activity in pauciarticular-onset juvenile rheumatoid arthritis. *Arthritis Rheum* 43 628–637. 10.1002/1529-0131(200003)43:3<628::aid-anr20>3.0.co;2-x 10728757

[B31] Fuchs T. A. Abed U. Goosmann C. Hurwitz R. Schulze I. Wahn V. (2007). Novel cell death program leads to neutrophil extracellular traps. *J. Cell Biol.* 176 231–241. 10.1083/jcb.200606027 17210947 PMC2063942

[B32] Gonzalez L. L. Garrie K. Turner M. D. (2020). Role of S100 proteins in health and disease. *Biochim. Biophys. Acta Mol. Cell Res.* 1867:118677. 10.1016/j.bbamcr.2020.118677 32057918

[B33] Goyette J. Geczy C. L. (2011). Inflammation-associated S100 proteins: new mechanisms that regulate function. *Amino Acids* 41 821–842. 10.1007/s00726-010-0528-0 20213444

[B34] Grebhardt S. Veltkamp C. Strobel P. Mayer D. (2012). Hypoxia and HIF-1 increase S100A8 and S100A9 expression in prostate cancer. *Int. J. Cancer* 131 2785–2794. 10.1002/ijc.27591 22505354

[B35] Grimbaldeston M. A. Geczy C. L. Tedla N. Finlay-Jones J. J. Hart P. H. (2003). S100A8 induction in keratinocytes by ultraviolet A irradiation is dependent on reactive oxygen intermediates. *J. Invest. Dermatol.* 121 1168–1174. 10.1046/j.1523-1747.2003.12561.x 14708622

[B36] Hanna R. N. Carlin L. M. Hubbeling H. G. Nackiewicz D. Green A. M. Punt J. A. (2011). The transcription factor NR4A1 (Nur77) controls bone marrow differentiation and the survival of Ly6C- monocytes. *Nat. Immunol.* 12 778–785. 10.1038/ni.2063 21725321 PMC3324395

[B37] Hausenloy D. J. Yellon D. M. (2016). Ischaemic conditioning and reperfusion injury. *Nat. Rev. Cardiol.* 13 193–209. 10.1038/nrcardio.2016.5 26843289

[B38] Healy A. M. Pickard M. D. Pradhan A. D. Wang Y. Chen Z. Croce K. (2006). Platelet expression profiling and clinical validation of myeloid-related protein-14 as a novel determinant of cardiovascular events. *Circulation* 113 2278–2284. 10.1161/circulationaha.105.607333 16682612

[B39] Heinemann A. S. Pirr S. Fehlhaber B. Mellinger L. Burgmann J. Busse M. (2017). In neonates S100A8/S100A9 alarmins prevent the expansion of a specific inflammatory monocyte population promoting septic shock. *FASEB J.* 31 1153–1164. 10.1096/fj.201601083r 27993995

[B40] Hessian P. A. Edgeworth J. Hogg N. (1993). MRP-8 and MRP-14, two abundant Ca(2+)-binding proteins of neutrophils and monocytes. *J. Leukoc. Biol.* 53 197–204. 10.1002/jlb.53.2.197 8445331

[B41] Heusch G. Gersh B. J. (2017). The pathophysiology of acute myocardial infarction and strategies of protection beyond reperfusion: a continual challenge. *Eur. Heart J.* 38 774–784. 27354052 10.1093/eurheartj/ehw224

[B42] Hilgendorf I. Gerhardt L. M. Tan T. C. Winter C. Holderried T. A. Chousterman B. G. (2014). Ly-6Chigh monocytes depend on Nr4a1 to balance both inflammatory and reparative phases in the infarcted myocardium. *Circ. Res.* 114 1611–1622. 10.1161/circresaha.114.303204 24625784 PMC4017349

[B43] Hobbs J. A. May R. Tanousis K. McNeill E. Mathies M. Gebhardt C. (2003). Myeloid cell function in MRP-14 (S100A9) null mice. *Mol. Cell. Biol.* 23 2564–2576. 10.1128/mcb.23.7.2564-2576.2003 12640137 PMC150714

[B44] Horckmans M. Ring L. Duchene J. Santovito D. Schloss M. J. Drechsler M. (2017). Neutrophils orchestrate post-myocardial infarction healing by polarizing macrophages towards a reparative phenotype. *Eur. Heart J.* 38 187–197. 28158426 10.1093/eurheartj/ehw002

[B45] Inaba H. Hokamura K. Nakano K. Nomura R. Katayama K. Nakajima A. (2009). Upregulation of S100 calcium-binding protein A9 is required for induction of smooth muscle cell proliferation by a periodontal pathogen. *FEBS Lett.* 583 128–134. 10.1016/j.febslet.2008.11.036 19059406

[B46] Ingersoll M. A. Spanbroek R. Lottaz C. Gautier E. L. Frankenberger M. Hoffmann R. (2010). Comparison of gene expression profiles between human and mouse monocyte subsets. *Blood* 115 e10–e19. 19965649 10.1182/blood-2009-07-235028 PMC2810986

[B47] Ionita M. G. Vink A. Dijke I. E. Laman J. D. Peeters W. van der Kraak P. H. (2009). High levels of myeloid-related protein 14 in human atherosclerotic plaques correlate with the characteristics of rupture-prone lesions. *Arterioscler. Thromb. Vasc. Biol.* 29 1220–1227. 10.1161/atvbaha.109.190314 19520974

[B48] Kallberg E. Vogl T. Liberg D. Olsson A. Bjork P. Wikstrom P. (2012). S100A9 interaction with TLR4 promotes tumor growth. *PLoS One* 7:e34207. 10.1371/journal.pone.0034207 22470535 PMC3314596

[B49] Kalogeris T. Baines C. P. Krenz M. Korthuis R. J. (2016). Ischemia/Reperfusion. *Compr. Physiol.* 7 113–170. 28135002 10.1002/cphy.c160006 PMC5648017

[B50] Katashima T. Naruko T. Terasaki F. Fujita M. Otsuka K. Murakami S. (2010). Enhanced expression of the S100A8/A9 complex in acute myocardial infarction patients. *Circ. J.* 74 741–748. 10.1253/circj.cj-09-0564 20190427

[B51] Kawakami R. Katsuki S. Travers R. Romero D. C. Becker-Greene D. Passos L. S. A. (2020). S100A9-RAGE Axis Accelerates Formation of Macrophage-Mediated Extracellular Vesicle Microcalcification in Diabetes Mellitus. *Arterioscler. Thromb. Vasc. Biol.* 40 1838–1853. 10.1161/atvbaha.118.314087 32460581 PMC7377960

[B52] Kingsley M. K. Bhat B. V. Badhe B. A. Dhas B. B. Parija S. C. (2020). Narciclasine improves outcome in sepsis among neonatal rats via inhibition of calprotectin and alleviating inflammatory responses. *Sci. Rep.* 10:2947. 10.1038/s41598-020-59716-7 PMC7031385 32076015

[B53] Kraakman M. J. Lee M. K. Al-Sharea A. Dragoljevic D. Barrett T. J. Montenont E. (2017). Neutrophil-derived S100 calcium-binding proteins A8/A9 promote reticulated thrombocytosis and atherogenesis in diabetes. *J. Clin. Invest.* 127 2133–2147. 10.1172/jci92450 28504650 PMC5451242

[B54] Landers-Ramos R. Q. Sapp R. M. VandeWater E. Macko J. Robinson S. Wang Y. (2017). Investigating the extremes of the continuum of paracrine functions in CD34-/CD31+ CACs across diverse populations. *Am. J. Physiol. Heart Circ. Physiol.* 312 H162–H172. 27793853 10.1152/ajpheart.00342.2016 PMC5283912

[B55] Lesniak W. (2011). Epigenetic regulation of S100 protein expression. *Clin. Epigenetics* 2 77–83. 10.1007/s13148-011-0023-9 21949546 PMC3156319

[B56] Leukert N. Sorg C. Roth J. (2005). Molecular basis of the complex formation between the two calcium-binding proteins S100A8 (MRP8) and S100A9 (MRP14). *Biol. Chem.* 386 429–434. 10.1515/bc.2005.051 15927886

[B57] Leukert N. Vogl T. Strupat K. Reichelt R. Sorg C. Roth J. (2006). Calcium-dependent tetramer formation of S100A8 and S100A9 is essential for biological activity. *J. Mol. Biol.* 359 961–972. 10.1016/j.jmb.2006.04.009 16690079

[B58] Li Y. Chen B. Yang X. Zhang C. Jiao Y. Li P. (2019). S100a8/a9 signaling causes mitochondrial dysfunction and cardiomyocyte death in response to ischemic/reperfusion injury. *Circulation* 140 751–764. 10.1161/circulationaha.118.039262 31220942

[B59] Lin C. R. Wei T. Y. Tsai H. Y. Wu Y. T. Wu P. Y. Chen S. T. (2015). Glycosylation-dependent interaction between CD69 and S100A8/S100A9 complex is required for regulatory T-cell differentiation. *FASEB J.* 29 5006–5017. 10.1096/fj.15-273987 26296369

[B60] Maekawa Y. Anzai T. Yoshikawa T. Asakura Y. Takahashi T. Ishikawa S. (2002). Prognostic significance of peripheral monocytosis after reperfused acute myocardial infarction:a possible role for left ventricular remodeling. *J. Am. Coll. Cardiol.* 39 241–246. 10.1016/s0735-1097(01)01721-1 11788214

[B61] Manitz M. P. Horst B. Seeliger S. Strey A. Skryabin B. V. Gunzer M. (2003). Loss of S100A9 (MRP14) results in reduced interleukin-8-induced CD11b surface expression, a polarized microfilament system, and diminished responsiveness to chemoattractants *in vitro*. *Mol. Cell. Biol.* 23 1034–1043. 10.1128/mcb.23.3.1034-1043.2003 12529407 PMC140712

[B62] Marenholz I. Heizmann C. W. Fritz G. (2004). S100 proteins in mouse and man: from evolution to function and pathology (including an update of the nomenclature). *Biochem. Biophys. Res. Commun.* 322 1111–1122. 10.1016/j.bbrc.2004.07.096 15336958

[B63] Marinkovic G. Grauen Larsen H. Yndigegn T. Szabo I. A. Mares R. G. de Camp L. (2019). Inhibition of pro-inflammatory myeloid cell responses by short-term S100A9 blockade improves cardiac function after myocardial infarction. *Eur. Heart J.* 40 2713–2723. 10.1093/eurheartj/ehz461 31292614

[B64] Marinkovic G. Koenis D. de Camp L. Jablonowski R. Graber N. de Waard V. (2020). S100A9 links inflammation and repair in myocardial infarction. *Circ. Res.* 127 664–676. 10.1161/circresaha.120.315865 32434457

[B65] McCormick M. M. Rahimi F. Bobryshev Y. V. Gaus K. Zreiqat H. Cai H. (2005). S100A8 and S100A9 in human arterial wall. Implications for atherogenesis. *J. Biol. Chem.* 280 41521–41529. 10.1074/jbc.m509442200 16216873

[B66] Miyamoto S. Ueda M. Ikemoto M. Naruko T. Itoh A. Tamaki S. (2008). Increased serum levels and expression of S100A8/A9 complex in infiltrated neutrophils in atherosclerotic plaque of unstable angina. *Heart* 94 1002–1007. 10.1136/hrt.2007.121640 18308864

[B67] Moore B. W. (1965). A soluble protein characteristic of the nervous system. *Biochem. Biophys. Res. Commun.* 19 739–744. 10.1016/0006-291x(65)90320-7 4953930

[B68] Morrow D. A. Wang Y. Croce K. Sakuma M. Sabatine M. S. Gao H. (2008). Myeloid-related protein 8/14 and the risk of cardiovascular death or myocardial infarction after an acute coronary syndrome in the Pravastatin or Atorvastatin Evaluation and Infection Therapy: thrombolysis in Myocardial Infarction (PROVE IT-TIMI 22) trial. *Am. Heart J.* 155 49–55. 10.1016/j.ahj.2007.08.018 18082488 PMC2645040

[B69] Muller I. Vogl T. Kuhl U. Krannich A. Banks A. Trippel T. (2020). Serum alarmin S100A8/S100A9 levels and its potential role as biomarker in myocarditis. *ESC Heart Fail.* 7 1442–1451. 10.1002/ehf2.12760 32462801 PMC7373886

[B70] Muller I. Vogl T. Pappritz K. Miteva K. Savvatis K. Rohde D. (2017). Pathogenic role of the damage-associated molecular patterns S100A8 and S100A9 in Coxsackievirus B3-Induced Myocarditis. *Circ. Heart Fail.* 10:e004125. 10.1161/CIRCHEARTFAILURE.117.004125 29158436

[B71] Nagareddy P. R. Kraakman M. Masters S. L. Stirzaker R. A. Gorman D. J. Grant R. W. (2014). Adipose tissue macrophages promote myelopoiesis and monocytosis in obesity. *Cell Metab.* 19 821–835. 10.1016/j.cmet.2014.03.029 24807222 PMC4048939

[B72] Nagareddy P. R. Murphy A. J. Stirzaker R. A. Hu Y. Yu S. Miller R. G. (2013). Hyperglycemia promotes myelopoiesis and impairs the resolution of atherosclerosis. *Cell Metab.* 17 695–708. 10.1016/j.cmet.2013.04.001 23663738 PMC3992275

[B73] Nahrendorf M. Swirski F. K. Aikawa E. Stangenberg L. Wurdinger T. Figueiredo J. L. (2007). The healing myocardium sequentially mobilizes two monocyte subsets with divergent and complementary functions. *J. Exp. Med.* 204 3037–3047. 10.1084/jem.20070885 18025128 PMC2118517

[B74] Neumann F. J. Ott I. Gawaz M. Richardt G. Holzapfel H. Jochum M. (1995). Cardiac release of cytokines and inflammatory responses in acute myocardial infarction. *Circulation* 92 748–755. 10.1161/01.cir.92.4.748 7543831

[B75] New S. E. Goettsch C. Aikawa M. Marchini J. F. Shibasaki M. Yabusaki K. (2013). Macrophage-derived matrix vesicles: an alternative novel mechanism for microcalcification in atherosclerotic plaques. *Circ. Res.* 113 72–77. 10.1161/circresaha.113.301036 23616621 PMC3703850

[B76] Newton K. Dixit V. M. (2012). Signaling in innate immunity and inflammation. *Cold Spring Harb. Perspect. Biol.* 4:a006049. 10.1101/cshperspect.a006049 PMC3282411 22296764

[B77] Odink K. Cerletti N. Bruggen J. Clerc R. G. Tarcsay L. Zwadlo G. (1987). Two calcium-binding proteins in infiltrate macrophages of rheumatoid arthritis. *Nature* 330 80–82. 10.1038/330080a0 3313057

[B78] Okada K. Arai S. Itoh H. Adachi S. Hayashida M. Nakase H. (2016). CD68 on rat macrophages binds tightly to S100A8 and S100A9 and helps to regulate the cells’ immune functions. *J. Leukoc. Biol.* 100 1093–1104. 10.1189/jlb.2a0415-170rrr 27312849

[B79] Otsuka K. Terasaki F. Ikemoto M. Fujita S. Tsukada B. Katashima T. (2009). Suppression of inflammation in rat autoimmune myocarditis by S100A8/A9 through modulation of the proinflammatory cytokine network. *Eur. J. Heart Fail.* 11 229–237. 10.1093/eurjhf/hfn049 19151078 PMC2645050

[B80] Passey R. J. Williams E. Lichanska A. M. Wells C. Hu S. Geczy C. L. (1999). A null mutation in the inflammation-associated S100 protein S100A8 causes early resorption of the mouse embryo. *J. Immunol.* 163 2209–2216. 10438963

[B81] Pei X. M. Tam B. T. Sin T. K. Wang F. F. Yung B. Y. Chan L. W. (2016). S100A8 and S100A9 are associated with doxorubicin-induced cardiotoxicity in the heart of diabetic mice. *Front. Physiol.* 7:334. 10.3389/fphys.2016.00334 PMC4974484 27547188

[B82] Petersen B. Wolf M. Austermann J. van Lent P. Foell D. Ahlmann M. (2013). The alarmin Mrp8/14 as regulator of the adaptive immune response during allergic contact dermatitis. *EMBO J.* 32 100–111. 10.1038/emboj.2012.309 23188082 PMC3545303

[B83] Prabhu S. D. Frangogiannis N. G. (2016). The biological basis for cardiac repair after myocardial infarction: from inflammation to fibrosis. *Circ. Res.* 119 91–112. 10.1161/circresaha.116.303577 27340270 PMC4922528

[B84] Pruenster M. Kurz A. R. Chung K. J. Cao-Ehlker X. Bieber S. Nussbaum C. F. (2015). Extracellular MRP8/14 is a regulator of beta2 integrin-dependent neutrophil slow rolling and adhesion. *Nat. Commun.* 6:6915. 10.1038/ncomms7915 PMC4411303 25892652

[B85] Pruenster M. Vogl T. Roth J. Sperandio M. (2016). S100A8/A9: from basic science to clinical application. *Pharmacol. Ther.* 167 120–131. 10.1016/j.pharmthera.2016.07.015 27492899

[B86] Rahimi F. Hsu K. Endoh Y. Geczy C. L. (2005). FGF-2, IL-1beta and TGF-beta regulate fibroblast expression of S100A8. *FEBS J.* 272 2811–2827. 10.1111/j.1742-4658.2005.04703.x 15943814

[B87] Rammes A. Roth J. Goebeler M. Klempt M. Hartmann M. Sorg C. (1997). Myeloid-related protein (MRP) 8 and MRP14, calcium-binding proteins of the S100 family, are secreted by activated monocytes via a novel, tubulin-dependent pathway. *J. Biol. Chem.* 272 9496–9502. 10.1074/jbc.272.14.9496 9083090

[B88] Raymond E. Dalgleish A. Damber J. E. Smith M. Pili R. (2014). Mechanisms of action of tasquinimod on the tumour microenvironment. *Cancer Chemother. Pharmacol.* 73 1–8. 10.1007/s00280-013-2321-8 24162378 PMC3889691

[B89] Reed G. W. Rossi J. E. Cannon C. P. (2017). Acute myocardial infarction. *Lancet* 389 197–210. 27502078 10.1016/S0140-6736(16)30677-8

[B90] Ryckman C. Vandal K. Rouleau P. Talbot M. Tessier P. A. (2003). Proinflammatory activities of S100: proteins S100A8, S100A9, and S100A8/A9 induce neutrophil chemotaxis and adhesion. *J. Immunol.* 170 3233–3242. 10.4049/jimmunol.170.6.3233 12626582

[B91] Sager H. B. Heidt T. Hulsmans M. Dutta P. Courties G. Sebas M. (2015). Targeting interleukin-1beta reduces leukocyte production after acute myocardial infarction. *Circulation* 132 1880–1890. 10.1161/circulationaha.115.016160 26358260 PMC4651795

[B92] Santamaria-Kisiel L. Rintala-Dempsey A. C. Shaw G. S. (2006). Calcium-dependent and -independent interactions of the S100 protein family. *Biochem. J.* 396 201–214. 10.1042/bj20060195 16683912 PMC1462724

[B93] Schafer B. W. Heizmann C. W. (1996). The S100 family of EF-hand calcium-binding proteins: functions and pathology. *Trends Biochem. Sci.* 21 134–140. 10.1016/s0968-0004(96)80167-8 8701470

[B94] Schelbergen R. F. de Munter W. van den Bosch M. H. Lafeber F. P. Sloetjes A. Vogl T. (2016). Alarmins S100A8/S100A9 aggravate osteophyte formation in experimental osteoarthritis and predict osteophyte progression in early human symptomatic osteoarthritis. *Ann. Rheum Dis.* 75 218–225. 10.1136/annrheumdis-2014-205480 25180294

[B95] Schiopu A. Cotoi O. S. (2013). S100A8 and S100A9: DAMPs at the crossroads between innate immunity, traditional risk factors, and cardiovascular disease. *Mediators Inflamm.* 2013:828354. 10.1155/2013/828354 PMC3881579 24453429

[B96] Scott N. R. Swanson R. V. Al-Hammadi N. Domingo-Gonzalez R. Rangel-Moreno J. Kriel B. A. (2020). S100A8/A9 regulates CD11b expression and neutrophil recruitment during chronic tuberculosis. *J. Clin. Invest.* 130 3098–3112. 10.1172/jci130546 32134742 PMC7259997

[B97] Shi S. Yi J. L. (2018). S100A8/A9 promotes MMP-9 expression in the fibroblasts from cardiac rupture after myocardial infarction by inducing macrophages secreting TNFalpha. *Eur. Rev. Med. Pharmacol. Sci.* 22 3925–3935. 29949169 10.26355/eurrev_201806_15278

[B98] Sreejit G. Abdel-Latif A. Athmanathan B. Annabathula R. Dhyani A. Noothi S. K. (2020). Neutrophil-Derived S100A8/A9 amplify granulopoiesis after myocardial infarction. *Circulation* 141 1080–1094. 10.1161/circulationaha.119.043833 31941367 PMC7122461

[B99] Sunahori K. Yamamura M. Yamana J. Takasugi K. Kawashima M. Yamamoto H. (2006). The S100A8/A9 heterodimer amplifies proinflammatory cytokine production by macrophages via activation of nuclear factor kappa B and p38 mitogen-activated protein kinase in rheumatoid arthritis. *Arthritis Res. Ther.* 8:R69. 10.1186/ar1939 PMC1526633 16613612

[B100] Tardif M. R. Chapeton-Montes J. A. Posvandzic A. Page N. Gilbert C. Tessier P. A. (2015). Secretion of S100A8, S100A9, and S100A12 by neutrophils involves reactive oxygen species and potassium efflux. *J. Immunol. Res.* 2015:296149. 10.1155/2015/296149 PMC4736198 27057553

[B101] Terry R. L. Miller S. D. (2014). Molecular control of monocyte development. *Cell. Immunol.* 291 16–21. 10.1016/j.cellimm.2014.02.008 24709055 PMC4162862

[B102] Urban C. F. Ermert D. Schmid M. Abu-Abed U. Goosmann C. Nacken W. (2009). Neutrophil extracellular traps contain calprotectin, a cytosolic protein complex involved in host defense against Candida albicans. *PLoS Pathog.* 5:e1000639. 10.1371/journal.ppat.1000639 19876394 PMC2763347

[B103] Vandal K. Rouleau P. Boivin A. Ryckman C. Talbot M. Tessier P. A. (2003). Blockade of S100A8 and S100A9 suppresses neutrophil migration in response to lipopolysaccharide. *J. Immunol.* 171 2602–2609. 10.4049/jimmunol.171.5.2602 12928412

[B104] Veglia F. Perego M. Gabrilovich D. (2018). Myeloid-derived suppressor cells coming of age. *Nat. Immunol.* 19 108–119. 10.1038/s41590-017-0022-x 29348500 PMC5854158

[B105] Voganatsi A. Panyutich A. Miyasaki K. T. Murthy R. K. (2001). Mechanism of extracellular release of human neutrophil calprotectin complex. *J. Leukoc. Biol.* 70 130–134. 11435495

[B106] Vogel B. Claessen B. E. Arnold S. V. Chan D. Cohen D. J. Giannitsis E. (2019). ST-segment elevation myocardial infarction. *Nat. Rev. Dis. Primers* 5:39. 10.1038/s41572-019-0090-3 31171787

[B107] Vogl T. Leukert N. Barczyk K. Strupat K. Roth J. (2006). Biophysical characterization of S100A8 and S100A9 in the absence and presence of bivalent cations. *Biochim Biophys Acta* 1763 1298–1306. 10.1016/j.bbamcr.2006.08.028 17050004

[B108] Vogl T. Ludwig S. Goebeler M. Strey A. Thorey I. S. Reichelt R. (2004). MRP8 and MRP14 control microtubule reorganization during transendothelial migration of phagocytes. *Blood* 104 4260–4268. 10.1182/blood-2004-02-0446 15331440

[B109] Vogl T. Stratis A. Wixler V. Voller T. Thurainayagam S. Jorch S. K. (2018). Autoinhibitory regulation of S100A8/S100A9 alarmin activity locally restricts sterile inflammation. *J. Clin. Invest.* 128 1852–1866. 10.1172/jci89867 29611822 PMC5919817

[B110] Vogl T. Tenbrock K. Ludwig S. Leukert N. Ehrhardt C. van Zoelen M. A. (2007). Mrp8 and Mrp14 are endogenous activators of Toll-like receptor 4, promoting lethal, endotoxin-induced shock. *Nat. Med.* 13 1042–1049. 10.1038/nm1638 17767165

[B111] Volz H. C. Laohachewin D. Seidel C. Lasitschka F. Keilbach K. Wienbrandt A. R. (2012). S100A8/A9 aggravates post-ischemic heart failure through activation of RAGE-dependent NF-kappaB signaling. *Basic Res. Cardiol.* 107:250. 10.1007/s00395-012-0250-z 22318783

[B112] Vora A. N. Bonaca M. P. Ruff C. T. Jarolim P. Murphy S. Croce K. (2012). Diagnostic evaluation of the MRP-8/14 for the emergency assessment of chest pain. *J. Thromb. Thrombolysis* 34 229–234. 10.1007/s11239-012-0705-y 22446997 PMC4113032

[B113] Wache C. Klein M. Ostergaard C. Angele B. Hacker H. Pfister H. W. (2015). Myeloid-related protein 14 promotes inflammation and injury in meningitis. *J. Infect. Dis.* 212 247–257. 10.1093/infdis/jiv028 25605866

[B114] Walters A. M. Porter G. A. Jr. Brookes P. S. (2012). Mitochondria as a drug target in ischemic heart disease and cardiomyopathy. *Circ. Res.* 111 1222–1236. 10.1161/circresaha.112.265660 23065345 PMC3507431

[B115] Wan E. Yeap X. Y. Dehn S. Terry R. Novak M. Zhang S. (2013). Enhanced efferocytosis of apoptotic cardiomyocytes through myeloid-epithelial-reproductive tyrosine kinase links acute inflammation resolution to cardiac repair after infarction. *Circ. Res.* 113 1004–1012. 10.1161/circresaha.113.301198 23836795 PMC3840464

[B116] Wang S. Song R. Wang Z. Jing Z. Wang S. Ma J. (2018). S100A8/A9 in Inflammation. *Front. Immunol.* 9:1298. 10.3389/fimmu.2018.01298 29942307 PMC6004386

[B117] Wang Y. Fang C. Gao H. Bilodeau M. L. Zhang Z. Croce K. (2014). Platelet-derived S100 family member myeloid-related protein-14 regulates thrombosis. *J. Clin. Invest.* 124 2160–2171. 10.1172/jci70966 24691441 PMC4001535

[B118] Wei X. Wu B. Zhao J. Zeng Z. Xuan W. Cao S. (2015). Myocardial Hypertrophic Preconditioning Attenuates Cardiomyocyte Hypertrophy and Slows Progression to Heart Failure Through Upregulation of S100A8/A9. *Circulation* 131 1506–1517. 10.1161/circulationaha.114.013789 25820336 PMC4415966

[B119] Wu Y. Li Y. Zhang C. Xi A. Wang Y. Cui W. (2014). S100a8/a9 released by CD11b+Gr1+ neutrophils activates cardiac fibroblasts to initiate angiotensin II-Induced cardiac inflammation and injury. *Hypertension* 63 1241–1250. 10.1161/hypertensionaha.113.02843 24711518

[B120] Yao D. Brownlee M. (2010). Hyperglycemia-induced reactive oxygen species increase expression of the receptor for advanced glycation end products (RAGE) and RAGE ligands. *Diabetes* 59 249–255. 10.2337/db09-0801 19833897 PMC2797929

[B121] Zimmer D. B. Eubanks J. O. Ramakrishnan D. Criscitiello M. F. (2013). Evolution of the S100 family of calcium sensor proteins. *Cell Calcium* 53 170–179. 10.1016/j.ceca.2012.11.006 23246155

[B122] Zou X. Sorenson B. S. Ross K. F. Herzberg M. C. (2013). Augmentation of epithelial resistance to invading bacteria by using mRNA transfections. *Infect. Immun.* 81 3975–3983. 10.1128/iai.00539-13 23940207 PMC3811834

